# Crying in Middle Childhood: A Report on Gender Differences

**DOI:** 10.1007/s11199-012-0136-4

**Published:** 2012-03-06

**Authors:** Francine C. Jellesma, Ad J. J. M. Vingerhoets

**Affiliations:** 1 Research Institute Child Development and Education, University of Amsterdam, PO 94208, 1090 GE Amsterdam, The Netherlands; 2 Clinical and Developmental Psychology, Tilburg University, Tilburg, The Netherlands

**Keywords:** Tears, Crying, Children, Emotion expression, Gender, Development

## Abstract

The aims of this study were (1) to confirm gender differences in crying in middle childhood and (2) to identify factors that may explain why girls cry more than boys in a Dutch sample (North Holland and Utrecht). We examined 186 children’s (age: 9–13 years) self-reports on crying, catharsis, seeking support for feelings, and internalizing feelings. Girls reported a greater crying frequency and crying proneness, and more emotional and physical catharsis after crying. In addition, they more frequently sought support for feelings and more often experienced sadness and somatic complaints than boys. Seeking help for negative feelings and the experience of sadness and somatic complaints were positively associated with crying frequency and crying proneness. Emotional catharsis was positively linked to crying proneness. Support was found for the potential mediating role of sadness and somatic complaints with respect to the gender difference in crying frequency and for the potential mediating role of emotional catharsis and somatic complaints for crying proneness. This study demonstrates that gender differences in crying frequency already exist in middle childhood and the findings suggest a linkage between these gender differences in crying and psychosocial factors.

## Introduction

“Fer crying out loud- there is a sex difference” wrote Lombardo and colleagues in 1983 (Lombardo et al. 1983) and, nearly 20 years later, a subsequent article was entitled: “For crying out loud—The differences persist into the ‘90s” (Lombardo et al. 2001). Both papers demonstrated a gender difference in crying in the United States, with (adult) females crying more frequently than males. Other studies, using different research methods, have yielded very comparable findings (see Vingerhoets and Scheirs 2000 for an overview) and a recent study, conducted in 37 countries, revealed that this gender differential in crying is a world-wide phenomenon, although the size of the difference varies considerably among countries (van Hemert et al. 2011).

Surprisingly, little is known about the determinants of this gender differential in crying frequency and it is also not clear why and when it develops. Crying in general, but in childhood in particular, is a largely neglected area of research. The present study builds upon the scarce previous published studies on this topic by exploring crying in middle childhood in a Dutch sample and by focusing on variables that might be possible determinants of the gender difference. More insight into the development and the potential mediators of gender differences in crying adds to our theoretical understanding of gender specific emotion expression and of the socio-emotional development of both genders.

Until now, it is unclear at what age the difference in (reported) crying frequency between men and women emerges. Frey and Langseth (1985) claims that girls cry more than boys from about age 13 onwards, due to menarche and its associated hormonal changes. More specifically, this author attributes the more frequent crying of girls to increases in the levels of the hormone prolactin. However, this notion is seriously challenged by some recent research findings. A Dutch study comparing the crying frequency of same age, menstruating and non-menstruating, girls failed to demonstrate the predicted differences in crying proneness and frequency (Van Tilburg et al. 2002). In addition, although there is some evidence suggesting that, in infancy boys may cry more often than girls (USA; Landreth 1941), this difference does not necessarily imply that boy infants also have a lower threshold for crying than girls. Perhaps, male infants have a stronger exploratory drive and put themselves more often in daring situations which may result in experiencing pain and frustration, both key antecedents of child crying.

Some support for this assumption is found by a Canadian study of Campbell and Eaton (1999) that showed that male infants have a higher activity level than female infants. Interestingly, in support of this speculation and *inconsistent* with the idea that boys cry more readily, exposing 11-month old babies to a standardized arm restraint procedure elicited crying in girls more quickly than in boys in European American, Chinese, and Japanese infants (Camras et al. 1998). In other words, maybe the genderdifferences in *crying proneness* (or crying threshold) do exist from birth on. However, more important is that these findings highlight that a seemingly simple outcome measure like the frequency of crying is not easy and unambiguously to interpret. Rather it may reflect a joint function of innate reactivity (or crying threshold), frequency of exposure to stressful situations, and self-regulatory capacity (Rottenberg and Vingerhoets in press).

Bekker and Vingerhoets (2001) have introduced a model to examine and interpret group (and individual) differences in crying frequency, which acknowledges the complexity of the crying frequency measure. These authors propose that in order to obtain a better understanding of the precise nature of the differences in crying frequency, one needs to know more about (1) differences in exposure to emotional, crying-inducing situations; (2) differences in the appraisal of (emotional) stimuli; (3) differences in the (maybe biologically determined) crying threshold; and (4) differences in the degree to which one has control over one’s tears (probably due to social learning). This model thus suggests that both biological and (psycho)social or cultural influences together may lay at the basis of gender difference in crying.

The strong variations in magnitudes of the gender differences in crying among countries further support the notion that these gender differences also are at least partly determined by social and cultural factors (van Hemert et al. 2011). According to the theory of hegemonic masculinity (Goodey 1997), there is a culturally normative ideal of male behavior with childhood and adolescence as crucial stages in identity development. More specifically, whereas people may accept crying as a normal emotional expression of young children, these reactions may change as children grow older. According to reports of American parents, at age 12, boys receive less encouragement and more disapproval in response to crying than girls (Hastrup et al. 2001). Especially for boys, the general social norm is thus to remain in control over one’s emotions in any situation. In addition, a literature review shows that boys not being able to control their tears may be at increased risk of being teased (von Salisch 2001). It thus seems plausible that social factors also have an influence when the gender differences in crying frequency first manifest themselves. The present study is a first more systematic attempt to evaluate the role of some social variables as positive determinants of the gender differential in crying frequency in middle childhood.

**Fig. 1:**
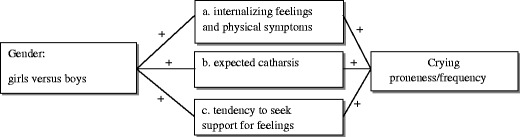
Assumed mediators of the gender difference in crying

A first potential mediator of the gender difference in crying that will be considered in this study is the prevalence of internalizing emotions and physical discomfort, which are important antecedents of crying. Internalizing problems refer to moods and emotions that children deal with internally (such as depression or, subclinically, feelings of helplessness, sadness, fear/anxiety or somatic complaints) rather than with dysregulations in behavior. They also are referred to as “overcontrolled” or “overinhibited” problems (Achenbach 1982). Since crying is strongly associated with feelings of helplessness and powerlessness (Frijda et al. 1989), it can be argued that crying is typically associated with internalizing feelings rather than with feelings such as anger. Children in the USA are generally socialized in a way that discourages the expression of internalizing feelings in boys and encourages male achievement, control, and power (Garside and Klimes-Dougan 2002; Parmley and Cunningham 2008). Cross-culturally, girls show more internalizing feelings (operationalized as withdrawal, somatic complaints, anxiety and depression) than boys with increasing age, i.e. across 6 to 17 year-olds (Crijnen et al. 1997). At least in the Netherlands, both parent reports and children’s self-reports show that boys have fewer internalizing problems than Dutch girls in middle childhood (van de Looij-Jansen et al. 2011). In the present study we focused on internalizing emotional feelings (sadness and fear/anxiety) and somatic complaints. Somatic complaints such as headaches, fatigue, and stomach aches in childhood are considered to be indicators of long-term negative emotional states and emotional disturbance in the USA (Robinson et al. 1988) and in the Netherlands (Meerum Terwogt et al. 2006). In addition, pain and injuries are important elicitors of crying in children, at least until the age of 16 (Rottenberg and Vingerhoets in press). This first hypothesis of possible mediation of the gender difference in crying by internalizing feelings and physical symptoms is depicted in Fig. 1 (a).

Secondly, differences in crying frequency may be associated with attitudes and implicit theories on the functions of crying and its possible consequences. One clear example of an attitude is the belief in the cathartic effect of crying (i.e., that crying brings relief and mood improvement; Rottenberg et al. 2008). Research in Dutch adults has demonstrated that women are more likely than men to report that they would express internalizing emotions because of the supposed cathartic effect (Timmers et al. 1998). In middle childhood, boys also have less positive expectations than girls about the outcome of the expression of sadness and are less inclined to express this emotion (USA; Fuchs and Thelen 1988). We thus hypothesize that another potential mediator of the crying differential in middle childhood is the belief in the cathartic powers of crying (depicted in Fig. 1, b).

Finally, in this specific age period, children’s coping repertoire develops and becomes more differentiated including both emotion focused and problem focused coping strategies to deal with everyday challenges and stressors (for a review see: Fields and Prinz 1997). Consequently, children no longer unconditionally rely on their caregivers. One specific coping strategy that is applied more often at this age in comparison to younger children is seeking social support from peers, when experiencing negative feelings (Fields and Prinz 1997). Yet, whereas it can be positive for both genders to communicate about their emotions, Dutch research demonstrated that it is not favorable for boys to ask support from peers who do not consider them as their best friend (Jellesma et al. 2008) and that boys tend to seek less support for their feelings than girls (de Boo and Wicherts 2009). Perhaps boys cry less frequently than girls partly because they are less likely to expect support from others (see also Vingerhoets and Scheirs 2000). This third potential mediator is depicted in Fig. 1(c).

Until now, few studies have specifically addressed the question when the gender differential in crying becomes manifest. In 1988 psychologists and social workers (USA) were requested to estimate at which age the gender differential would emerge. The predicted mean age was 8.4 years (Hastrup et al. 2001). In subsequent studies this estimate, however, failed to receive empirical support from two US studies. Parental reports of children’s crying frequency during 1 week in the summer break revealed no clear gender differences in 1- to 12-year-olds (Hastrup et al. 2001). Using a broader age range, the same researchers found that the gender differential in self-monitored 2 week crying frequency (in the summer period) emerged around age 13. This was confirmed in a UK study based on parents’ global estimates of crying frequency, which also suggested the differential to emerge after primary school, after the age of 12 (Shepherd et al. 1971). These findings thus seem to support the hypothesis that the development of the gender differential in crying is linked to the onset of adolescence, probably resulting from the close interaction between biological and psychological developments. Major limitations of these studies were the small sample sizes and recordings made by parents during the summer vacation period, when children perhaps are exposed to less peer pressure than when at school. Further, in this period, the children may have more opportunities to share their emotions with parents. In middle childhood, children still genuinely express their emotions in the presence of their parents, who provide trust and support (von Salisch 2001).

A more recent Dutch study with a large sample size, in contrast, showed that the crying differential was present already in the self-reports –collected at school- of 11-year-old children, which was the youngest age group in the sample (Van Tilburg et al. 2002). Same age boys reported a significant lower 4 week crying frequency and crying proneness than girls.

How can these dissimilar research findings be explained? Western countries differ mutually considerably in the strength of the stereotyped, normative ideas about masculinity and femininity (van Hemert et al. 2011). For example, the Netherlands are considerably less ‘masculine’ compared to the USA and the UK (Hofstede’s masculinity index of 14 versus 62 and 66, respectively; Hofstede 2003), which means that in the Netherlands there is probably less social pressure and reinforcement of the traditional masculine role model, resulting in more equality between men and women. In this light, it is even more remarkable that Van Tilburg et al. (2002) found the gender differential to be present already in middle childhood in a Dutch sample. Yet, research among adults also showed that the gender difference in crying is more substantial in the Netherlands than in many non-western, collectivistic cultures (van Hemert et al. 2011). It could be argued that such a socio-cultural climate stimulates the expression of emotions and limits possible inhibitory forces on emotional disclosure. Furthermore, as stated above, the summer period might not be the best time to assess the gender differential in children from a social perspective.

### The Present Study

The aims of the current study were to replicate and extend the findings of Van Tilburg et al. (2002), which demonstrated that the gender differential in crying is already present before the age of 13 and to gain more insight into variables that might explain the gender differential. Similar to the Van Tilburg et al. study, the present study also focuses on crying proneness and previous-month estimates of crying frequencies of Dutch children. As has been done in previous studies (Lombardo et al. 2001; Van Tilburg et al. 2002), we also used self-report measures. Whereas crying frequency is considered to be determined predominantly by environmental factors and exposure to events, crying proneness is considered as a more stable personality trait. It is thus important to assess both variables.

We included a slightly younger age group (grades 6–8; i.e., age 9–13) compared to Van Tilburg et al., because they found that the gender differential was already present in their youngest age group (11 years). We expected to find the gender difference in crying and the potential mediating processes to be present in children in all grades.

In conclusion, based on the literature described above, we formulated the following hypotheses:

1. Girls report a higher 4 week crying frequency than boys
2. Girls report to be more prone to cry than boys
3. The gender differential in crying frequency and crying proneness is mediated by (see Fig. 1):
  - Girls experiencing more internalizing feelings and physical symptoms than boys
  - Girls believing more strongly that crying results in catharsis than boys
  - Girls seeking support for feelings more frequently than boys

## Method

### Participants and Procedure

Participants were 74 boys and 112 girls, aged 9–13: boys had a mean age of 10.5 (*SD* = 1.02) and girls 10.7 (*SD* = 1.04).

Third year students of Educational Sciences approached schools by telephone and, if the schools showed interest, they visited them to provide further information. Seven schools were approached, four of which expressed interested to participate. The schools were located in three cities: Amsterdam (two schools: North Holland), Mijdrecht (Utrecht) and IJmuiden (North Holland), in neighborhoods that are an adequate representation of the Dutch society.

Written parental informed consent was received for 75% of the children (*N* = 186) in response to information letters to the parents of grades 6–8. Two research-assistants were present to explain the questionnaires and answer any questions. It took 30–40 min for the children to complete the questionnaires. Data were collected during regular school hours in the classroom.

### Materials

The questions about crying were derived from the Adult Crying Inventory (ACI; Vingerhoets and Cornelius 2002).

**Crying Frequency**: children were asked to estimate the frequency of their crying episodes in the last 4 weeks. For validation purposes, children also rated their general crying frequency on a 5 point likert-scale (from (almost) never to very often. The significant, positive correlation (Spearman *r* = .62) indeed supports the validity of these self-reports. A natural log transformation was performed on this variable, because it was skewed with most children reporting no or very few crying episodes.

**Crying Proneness**: crying proneness was assessed using the short version of part A of the ACI containing 18 items describing different feelings and situations on a 5-point scale (*α* = .82): (almost) never/sometimes/every now and then/often/(almost) always. Situations irrelevant for the current age group were deleted (e.g., *I cry when making love*). One new item was added to the questionnaire*: I cry when I don’t get my way.* The items (including the original Dutch wording) are presented in Appendix I. Scale scores were created by averaging the individual item scores*.*

**Emotional and Physical Catharsis**: children indicated whether they generally feel emotionally (1 item) and physically (1 item) worse, the same, or better after a crying episode.

**Support for Feelings** was measured with the Support for Feelings subscale of the Children’s Coping Strategies Checklist (Ayers et al. 1996; de Boo and Wicherts 2009). This scale consists of 4 items on a 4-point Likert scale (never/sometimes/often/always) (e.g., *You tell people how you feel about the problem*). The internal consistency in the present sample was good, *α =* .82*.*

**Internalizing Feelings**: Two 4-item subscales of the Mood List (Sadness (example item: I feel sad, *α* = .76), and Anxiety/Fear (example item: I feel scared, *α* = .86)) were used to assess internalizing feelings. Items were scored on a 3-point scale (never/sometimes/often). Its validity has been established in previous research (Jellesma et al. 2006; Jellesma et al. 2007).

**Somatic Complaints** were assessed with the Somatic Complaint List (Jellesma et al. 2007). This list includes 11 somatic complaints (e.g., I feel tired, I have a headache) on a 5-point scale: (almost) never, very sometimes, sometimes, often, and very often. The internal consistency in this study was .78 and the validity has been supported in previous research (Jellesma et al. 2006; Jellesma et al. 2007).

Scale scores for the last three scales were created *by summing and averaging items pertaining to each scale.*

## Results

### Descriptives

The mean scores and standard deviations for boys and girls on all variables are presented in Table 1. A one way MANOVA applied to analyze whether there were gender differences on Emotional Catharsis, Physical Catharsis, Support for Feelings, Sadness, Fear/Anxiety, and Somatic Complaints revealed a main effect for gender, *F*(6,177) = 2.19, *p* = .05. Post hoc *t*-tests confirmed the expected gender differences in all variables except for fear/anxiety (see Table 1). Compared to boys, girls reportedly experienced more emotional and physical catharsis, sought more support for feelings, and reported more sadness and somatic complaints. As a consequence, the variable fear/anxiety was not included in subsequent analyses.

**Table 1:** Means and standard deviations of the studied variables for boys and girls

Variable	Scale range	Boys		Girls		*t*
		*M*	*SD*	*M*	*SD*	*DF* = 184
Crying proneness	1–5	1.66_a_	.45	1.87_b_	.47	2.93**
Crying frequency	0-unlimited	2.92_a_	4.84	3.79_b_	4.47	2.68** (on ln transformed)
Emotional Catharsis	1–3	2.19_a_	.91	2.49_b_	.69	2.50**
Physical catharsis	1–3	2.12_a_	.81	2.32_b_	.72	1.79*
Support for feelings	1–4	1.97_a_	.65	2.14_b_	.64	1.76*
Sadness	1–3	1.56_a_	.56	1.72_b_	.54	1.88*
Fear/Anxiety	1–3	1.47_a_	.56	1.59_a_	.58	1.46
Somatic complaints	1–5	1.95_a_	.54	2.17_b_	.81	2.08*

*p < .05, **p < .01 (one sided)

### Gender Differential in Crying

Crying frequency was skewed with most children reporting no or very few crying episodes. We therefore performed a natural log transformation before including crying frequency as a dependent variable in an analysis of variance. Because our sample included children that were younger than the participants in Van Tilburg et al.’s sample, we performed a MANOVA with gender and grade as independent variables and with ln transformed crying frequency and crying proneness as dependent variables. Since this analysis yielded no significant gender x grade interaction, *F*(2, 176) = 2.13, *p* = .12, this interaction was removed from the model. As anticipated, we also did not find a main effect for grade, *F*(2,177) = 0.08, *p* = .93, whereas the expected main effect for gender was significant, *F*(2,177) = 5.23, *p* < .01. Girls reported a higher crying frequency (H1) and proneness (H2) than boys (see Table 1).

### Mediators of the Crying Differential

In Table 2, correlations between the potential mediators and crying frequency and proneness are presented. As hypothesized, positive associations were found between sadness, somatic complaints and seeking support for feelings, on the one hand, and crying frequency and proneness, on the other hand. However, physical catharsis was unrelated to crying and emotional catharsis was related to crying proneness, but not to crying frequency.

**Table 2:** Correlations between ln transformed crying frequency and crying proneness, catharsis, support for feelings, sadness and somatic complaints

	ln crying frequency	Crying proneness	Emotional catharsis	Physical catharsis	Support for feelings	Sadness	Somatic complaints
ln Crying Frequency	–	.40**	.11	.08	.15*	.24**	.31**
Crying Proneness	.32**/.42**	–	.20**	.14	.31**	.30**	.36**
Emotional Catharsis	−.01/.16	.20/.15	–	.44**	.07	.06	−.01
Physical catharsis	.05/.07	.12/.11	.38**/.47**	–	.11	.08	−.06
Support for feelings	.26*/.03	.43**/.21*	.03/.07	.02/.15	–	.22**	.18**
Sadness	.14/.28**	.23*/.31**	.04/.03	.04/.08	.23*/.18	–	.31**
Somatic Complaints	.26*/.31**	.34**/.35**	−.08/−.02	−.14/−.06	.10/.20*	.48**/.23*	–

* p < .05, ** *p* < .01

The lower diagonals of the table present the correlations for boys and girls separately (boys/girls)

To analyze the mediation effects further, we used a version of the Sobel test (Sobel 1982), which tests whether the indirect effect of gender on crying frequency or crying proneness (H3) through the mediators is significantly different from zero. With the bootstrap method of Preacher and Hayes (2004) especially recommended for skewed variables and small samples, the indirect effect and bias-corrected 95% confidence interval was estimated for each mediator and for all the mediators as a group, based on 1000 bootstrap samples using the available macro for SPSS. Crying frequency was not transformed for this analysis.

The results of this mediation analyses are summarized in Table 3. Significant mediators of the gender difference in crying frequency were sadness and somatic complaints, whereas for the gender difference in crying proneness, emotional catharsis and somatic complaints emerged as important mediators. This result can be considered as partial support for H3 stating that internalizing feelings mediate the gender differences in crying. The anticipated mediating effect of catharsis was not found for crying frequency, whereas partial support was obtained for crying proneness (H3b; only for emotional, but not for physical catharsis). The hypothesized mediating effect of seeking support for feelings (H3b) also failed to receive support.

**Table 3:** Bias corrected and accelerated 95% confidence intervals of the potential mediators of the gender crying differential

	Bias corrected and accelerated 95% confidence intervals	
Mediators	Independent variable gender and dependent variable crying frequency	Independent variable gender and dependent variable crying proneness
Emotional Catharsis	−1.03 to .06	.002 to .07
Physical catharsis	−.05 to .75	−.01 to .04
Support for feelings	−.04 to .51	−.06 to .06
Sadness	.07 to .63	−.05 to .06
Somatic complaints	.06 to .59	.08 to .09
Total	.04 to 1.01	.04 to .19

*R* ^2^ = .09, *F*(6, 177) = 3.01, *p* < .01 for Crying Frequency and *R* ^2^ = .27, *F*(6, 177) = 10.80, *p* < .01 for Crying Proneness

## Discussion

The aims of this study were twofold: (1) to establish gender differences in crying in a Dutch middle childhood sample and (2) to identify factors that might explain why girls cry more often than boys. We expected to confirm the gender differential in crying in this age group and that this difference in crying was related to girls experiencing more internalizing feelings and physical symptoms, seeking support for feelings more often and perceiving greater catharsis than boys.

Our results confirm that the gender difference in crying is indeed already present in middle childhood. Furthermore, it was demonstrated that girls reportedly experience more emotional and physical catharsis from crying than boys, more frequently seek support for feelings, and more often experience sadness and suffer from somatic complaints. Except for catharsis, all of these variables were positively related to crying frequency and crying proneness. Emotional catharsis did show a positive association with crying proneness. Support was found for the potential mediating role of sadness and somatic complaints with respect to the gender difference in crying frequency and for the potential mediating role of emotional catharsis and somatic complaints for crying proneness.

We thus found slightly different results for the estimates of crying frequency in the past month and crying proneness, which once more stresses the importance of distinguishing between these two variables in future studies as well. As explained in the Introduction, crying proneness can be thought of as more stable construct than crying frequency (Van Tilburg et al. 2002). Whereas children’s crying frequency estimations will probably have been influenced by their actual crying experiences in real life, their ratings of crying proneness more likely reflect their crying threshold, i.e., the probability that they will cry, if they would experience certain situations or feelings. Future research should provide more clarity about the possible interaction between crying proneness and exposure to real life events in predicting children’s crying frequency. In addition, the lack of a relationship between reported emotional catharsis and crying frequency suggests that even though children do not generally expect crying to help them feel better (rather, they believe that inhibition of crying facilitates recovery when feeling sad or distressed, cf. Stegge et al. 2004), they nevertheless can feel overwhelmed by a situation or expect that crying will help them feel better under certain circumstances (e.g., depending on who is with them). These and other potential explanations for the absence of a relationship between notions on emotional catharsis and crying frequency deserve further investigation.

We further expected mediation of the crying difference by psychological and physical state (sadness, fear/anxiety and somatic complaints), but this was only partially supported. More precisely, only level of somatic complaints was a significant potential mediator and sadness was significant for crying frequency. It may be worthwhile to study these effects again with a different operationalization of these emotional states. The Mood List (that was used to measure sadness) assesses common, everyday emotional states; perhaps stronger effects might be found when assessing factors that are more closely connected with crying in children and adolescents (e.g., frustration, physical pain).

The present findings as well as the results of recent research in the USA (Cassano and Zeman 2010) suggest that, in modern society, gender specific socialization (still) plays a role in children’s emotional functioning. Although some authors have suggested that changing gender roles may alter gender expectations of emotion expression (LaFrance and Banaji 1992), research in adults has shown that it may take a long time before these societal changes influence gender differences in emotion expression, such as crying (Lombardo et al. 2001). Alternatively, changes in society resulting in a more feminine, individualistic culture will not always necessarily lead to a decrease in gender differences in emotion expression. Van Hemert et al.’s (2011) study across 37 countries revealed that the gender differential is *most* pronounced in wealthy and individualistic Western countries. Perhaps, in the more masculine and collectivistic cultures, self-control and moderation are more valued, whereas, in more individualistic and feminine countries, emotion expression is more appreciated. In other words: females in modern Western countries may feel freer to express their emotions than females in non-Western countries.

More specific information about the potential influence of gender roles may be obtained from research that focuses on differences in the gender differential *within* cultures. Socialization of gender roles is to a great extent influenced by family processes. The majority of American parents still create different environments for sons and daughters and also interact differently with the two genders (Chaplin et al. 2005; Garside and Klimes-Dougan 2002; Parmley and Cunningham 2008). There are nevertheless also considerable differences between families within cultures in this respect, with some families holding a more feministic, less traditional attitude (Blakemore and Hill 2008). Within these families, American as well as Dutch children seem less affected by gender stereotypes, as indicated by their attitudes (Chaplin et al. 2005; Davis and Wills 2010; Ex and Janssens 1998) and self-reports of crying (in adolescence; Bronstein et al. 1996).

For the sake of completeness and to complicate things further, children also appear to apply different display rules, dependent on who is with them (USA; Zeman and Garber 1996). In the company of their parents, they may more likely cry than when in the presence of peers or strangers. Future research could perhaps further enlighten the role of socialization in explaining gender differences in crying by investigating the influence of parental gender role ideologies on boys’ and girls’ crying behavior and taking into account the presence of specific others. In order to arrive at a full understanding of crying, it must not be studied in a social vacuum, but there should be adequate attention for the whole context. Rosenwein (2002) proposes the existence of what she calls ‘emotional communities’ within a society, which each have their own emotional expression and specific display rules.

### Study Limitations

Major limitations of this study are twofold: (1) our reliance on self-report data and (2) the cross-sectional nature of the study. Shared method variance might have led to an inflation of the relationships between the studied variables. We had, however, valid reasons for using self-reports of crying. We were interested in the overall difference in general crying and not in crying to one specific event or stimulus. The use of a movie (cf., Sternbach 1962) was therefore no option, whereas it also is almost impossible to collect objective real-life information on crying, because of its relative infrequent occurrence (Scheirs and Sijtsma 2001). Self-reports on crying behavior are most often used in studies with adult participants (Lombardo et al. 2001; Van Hemert et al. 2011) which has been proven to be reliable and valid (Kraemer and Hastrup 1986; Labott et al. 1991) although *yearly* estimates might be not very accurate (Hastrup et al. 2001). The use of self-reports of children from the age of 8 on may be expected to be feasible and meaningful (Stone and Lemanek 1990), because, from that age on, children are able to report on their thoughts and feelings and to provide accurate information regarding diverse experiences and situations. Further, it has been suggested that the subjective experience of emotions and emotion expression, by definition, cannot be captured by physiological or other objective measures (Scheirs and Sijtsma 2001). In order to prevent memory biases to influence the self-reports, in future studies the use of diaries (cf., Bylsma et al. 2008) might be considered. One must, however, be aware of the possible disadvantages of this method as well. The detailed recording of a certain behavior might affect its occurrence and phenomenology. In addition, it may be very demanding for children to keep a diary over longer periods of time (Lyberg and Kasprzyk 1991).

The cross-sectional nature of the study prevents drawing definite conclusions about the precise nature of the relations between the measured variables. For example, it cannot be excluded that the children report greater sadness *because* they cry often or that both crying frequency and the experience of symptoms are connected to a third variable, such as neuroticism.

Crying, in particular of children and older individuals, is a neglected research topic in the behavioral sciences. Nevertheless, adequate insight into its developments with increasing age in terms of not only frequency, but in particular with respect to antecedents and *intra*- and *inter*individual effects might contribute significantly to our understanding of socio-emotional and maybe even moral development (Rottenberg and Vingerhoets in press; Vingerhoets 2012).

## Appendix I

Table 4

**Table 4:** Items used to measure crying proneness (short version of the Adult Crying Inventory part A)

Dutch	English
1. Ik huil als ik droevig ben.	I cry when I feel sad
2. Ik huil als ik me schaam.	I cry when I feel embarrassed
3. Ik huil als ik me gelukkig voel.	I cry when I feel happy
4. Ik huil als ik me opgelucht voel.	I cry when I feel relieved
5. Ik huil als ik me machteloos voel. (je hebt het gevoel dat je iets naars niet kunt veranderen)	I cry when I feel powerless (you feel like you cannot change something bad)
6. Ik huil als iemand mij beledigt of kwetst.	I cry when someone insults me
7. Ik huil als ik bang ben.	I cry when I feel anxious
8. Ik huil als ik kwaad ben.	I cry when I feel sad
9. Ik huil als ik iets zielig vindt voor iemand.	I cry when I pity someone
10. Ik huil als ik iets zielig of vervelend vindt voor mezelf.	I cry when I pity myself
11. Ik huil als ik pijn heb.	I cry when I am in pain
12. Ik huil als ik me wanhopig voel.	I cry when I feel desperate
13. Ik huil als ik me in de steek gelaten voel.	I cry when I feel abandoned
14. Ik huil als ik me schuldig voel.	I cry when I feel guilty
15. Ik huil als ik trots ben.	I cry when I feel proud
16. Ik huil als ik me eenzaam (alleen) voel.	I cry when I feel lonely
17. Ik huil als ik ontroerd ben (je vindt iets mooi of lief).	I cry when I feel moved (you find something beautiful or sweet)
18. Ik huil als ik mijn zin niet krijg.^a^	I cry when I do not get my way^a^

^a^This item was added for the current research

## References

[CR1] Achenbach TM (1982). Developmental psychopathology.

[CR2] Ayers TS, Sandler IN, West SG, Roosa MW (1996). A dispositional and situational assessment of children’s coping: Testing alternative models of coping. Journal of Personality.

[CR3] Bekker MHJ, Vingerhoets AJJM, Vingerhoets AJJM, Cornelius RR (2001). Male and female tears: Swallowing versus shedding? The relationship between crying, biological sex and gender. Adult crying: A biopsychosocial approach.

[CR4] Blakemore JEO, Hill CA (2008). The child gender socialization scale: A measure to compare traditional and feminist parents. Sex Roles.

[CR5] Bronstein P, Briones M, Brooks T, Cowan B (1996). Gender and family factors as predictors of late adolescent emotional expressiveness and adjustment: A longitudinal study. Sex Roles.

[CR6] Bylsma LM, Vingerhoets AJJM, Rottenberg J (2008). When is crying cathartic? an international study. Journal of Social and Clinical Psychology.

[CR7] Campbell DW, Eaton WO (1999). Sex differences in the activity levels of infants. Infant and Child Development.

[CR8] Camras LA, Oster H, Campos J, Campos R, Ujiie T, Miyake K, Wang L, Meng Z (1998). Production of emotional facial expressions in European American, Japanese, and Chinese infants. Journal of Personality and Social Psychology.

[CR9] Cassano MC, Zeman JL (2010). Parental socialization of sadness regulation in middle childhood: The role of expectations and gender. Developmental Psychology.

[CR10] Chaplin TM, Cole PM, Zahn-Waxler C (2005). Parental socialization of emotion expression: Gender differences and relations to child adjustment. Emotion.

[CR11] Crijnen AAM, Achenbach TM, Verhulst FC (1997). Comparisons of problems reported by parents of children in 12 cultures: Total problems, externalizing, and internalizing. Journal of the American Academy of Child & Adolescent Psychiatry.

[CR12] Davis SN, Wills JB (2010). Adolescent gender ideology socialization: Direct and moderating effects of fathers’ beliefs. Sociological Spectrum.

[CR13] de Boo GM, Wicherts J (2009). Assessing cognitive and behavioral coping strategies in children. Cognitive Therapy and Research.

[CR14] Ex C, Janssens J (1998). Maternal influences on daughters’ gender role attitudes. Sex Roles.

[CR15] Fields L, Prinz RJ (1997). Coping and adjustment during childhood and adolescence. Clinical Psychology Review.

[CR16] Frey WH, Langseth M (1985). Crying: The mystery of tears.

[CR17] Frijda NH, Kuipers P, Ter Schure E (1989). Relations among emotion, appraisal, and emotional action readiness. Journal of Personality and Social Psychology.

[CR18] Fuchs D, Thelen MH (1988). Children’s expected interpersonal consequences of communicating their affective state and reported likelihood of expression. Child Development.

[CR19] Garside RB, Klimes-Dougan B (2002). Socialization of discrete negative emotions: Gender differences and links with psychological distress. Sex Roles.

[CR20] Goodey J (1997). Boys don’t cry—masculinities, fear of crime and fearlessness. British Journal of Criminology.

[CR21] Hastrup JL, Kraemer DT, Bornstein RF, Trezza GR, Vingerhoets AJJM, Cornelius RR (2001). Crying frequency across the lifespan. Adult crying: A biopsychosocial approach.

[CR22] Hofstede GH (2003). Culture’s consequences: Comparing values, behaviors, institutions, and organizations across nations.

[CR23] Jellesma FC, Rieffe C, Terwogt MM, Kneepkens CMF (2006). Somatic complaints and health care use in children: Mood, emotion awareness and sense of coherence. Social Science & Medicine.

[CR24] Jellesma FC, Rieffe C, Terwogt MM (2007). The somatic complaint list: Validation of a self-report questionnaire assessing somatic complaints in children. Journal of Psychosomatic Research.

[CR25] Jellesma FC, Rieffe C, Terwogt MM (2008). My peers, my friend, and I: Peer interactions and somatic complaints in boys and girls. Social Science & Medicine.

[CR26] Kraemer DL, Hastrup JL (1986). Crying in natural settings: Global estimates, self-monitored frequencies, depression and sex-differences in an undergraduate population. Behaviour Research and Therapy.

[CR27] Labott SM, Martin RB, Eason PS, Berkey EY (1991). Social reactions to the expression of emotion. Cognition & Emotion.

[CR28] LaFrance M, Banaji M, Clark MS (1992). Toward a reconsideration of the gender-emotion relationship. Emotion and social behavior.

[CR29] Landreth C (1941). Factors associated with crying in young children at the nursery and at home. Child Development.

[CR30] Lombardo WK, Cretser GA, Lombardo B, Mathis SL (1983). Fer cryin’ out loud: There is a sex difference. Sex Roles.

[CR31] Lombardo WK, Cretser GA, Roesch SC (2001). For crying out loud—the differences persist into the ‘90s. Sex Roles.

[CR32] Lyberg LE, Kasprzyk D, Biemer PP, Groves RM, Lyberg LE, Mathiowetz NA, Sudman S (1991). Data collection methods and measurement error: An Overview. Measurement errors in surveys.

[CR33] Meerum Terwogt MM, Rieffe C, Miers AC, Jellesma FC, Tolland A (2006). Emotions and self-esteem as indicators of somatic complaints in children. Infant and Child Development.

[CR34] Parmley M, Cunningham JG (2008). Children’s gender-emotion stereotypes in the relationship of anger to sadness and fear. Sex Roles.

[CR35] Preacher KJ, Hayes AF (2004). SPSS and SAS procedures for estimating indirect effects in simple mediation models. Behavior Research Methods.

[CR36] Robinson DP, Greene JW, Walker LS (1988). Functional somatic complaints in adolescents: Relationship to negative life events, self-concept, and family characteristics. The Journal of Pediatrics.

[CR37] Rosenwein, B. H. (2002). Worrying about emotions in history. *The American Historical Review*, *107*, 821–845. Retrieved from http://www.jstor.org/stable/10.1086/532498 10.1086/532498 12132538

[CR38] Rottenberg, J., & Vingerhoets, A. J. J. M. (in press). Crying: Call for a life-span approach. *Social and Personality Psychology Compass*. doi:10.1111/j.1751-9004.2012.00426.x.

[CR39] Rottenberg J, Bylsma LM, Vingerhoets AJJM (2008). Is crying beneficial?. Current Directions in Psychological Science.

[CR40] Scheirs JGM, Sijtsma K, Vingerhoets AJJM, Cornelius RR (2001). The study of crying: Some methodological considerations and a comparison of methods for analyzing questionnaires. Adult crying: A biopsychosocial approach.

[CR41] Shepherd M, Oppenheim B, Mitchell S (1971). Childhood behaviour and mental health.

[CR42] Sobel ME (1982). Asymptotic confidence intervals for indirect effects in structural equation models. Sociological Methodology.

[CR43] Stegge H, Meerum Terwogt M, Reijntjes A, van Tijen N, Nyklicek I, Temoshok L, Vingerhoets AJJM (2004). Children’s conception of the emotion process: Consequences for emotion regulation. Emotional expression and health; advances in theory, assessment and clinical applications.

[CR44] Sternbach RA (1962). Assessing differential autonomic patterns in emotions. Journal of Psychosomatic Research.

[CR45] Stone WL, Lemanek KL, LaGreca AM (1990). Developmental issues in children’s self-reports. Through the eyes of the child: Obtaining self-reports from children and adolescents.

[CR46] Timmers M, Fischer AH, Manstead ASR (1998). Gender differences in motives for regulating emotions. Personality and Social Psychology Bulletin.

[CR47] van de Looij-Jansen PM, Jansen W, de Wilde EJ, Donker MCH, Verhulst FC (2011). Discrepancies between parent-child reports of internalizing problems among preadolescent children: Relationships with gender, ethnic background, and future internalizing problems. Journal of Early Adolescence.

[CR48] van Hemert DA, van de Vijver FJR, Vingerhoets AJJM (2011). Culture and crying: Prevalences and gender differences. Cross-Cultural Research.

[CR49] Van Tilburg MAL, Unterberg ML, Vingerhoets AJJM (2002). Crying during adolescence: The role of gender, menarche, and empathy. British Journal of Developmental Psychology.

[CR50] Vingerhoets, A. J. J. M. (2012). *Why Humans. Cry. Unraveling the Mysteries of Tears*. Oxford: Oxford University Press.

[CR51] Vingerhoets AJJM, Cornelius RR (2002). Adult crying: A biopsychosocial approach.

[CR52] Vingerhoets AJJM, Scheirs JGM, Fischer AD (2000). Sex differences in crying: Empirical findings and possible explanations. Gender and emotion: Social psychological perspectives.

[CR53] von Salisch M (2001). Children’s emotional development: Challenges in their relationships to parents, peers, and friends. International Journal of Behavioral Development.

[CR54] Zeman J, Garber J (1996). Display rules for anger, sadness, and pain: It depends on who is watching. Child Development.

